# Sentinels of inequity: examining policy requirements for equity-oriented primary healthcare

**DOI:** 10.1186/s12913-018-3501-3

**Published:** 2018-09-10

**Authors:** Josée G. Lavoie, Colleen Varcoe, C. Nadine Wathen, Marilyn Ford-Gilboe, Annette J. Browne, On behalf of the EQUIP Research Team

**Affiliations:** 1 Department of Community Health Sciences, Faculty of Medicine, University of Manitoba, Ongomiizwin Research, 715 John Buhler Research Centre, 727 McDermot Ave, Winnipeg, MB R3E 3P5 Canada; 2 School of Nursing, The University of British Columbia, T201-2211 Wesbrook Mall, Vancouver, BC V6T 2B5 Canada; 3 Centre for Research and Education on Violence against Women and Children, Faculty of Information & Media Studies, Western University, FIMS & Nursing Building, Room 2050, London, ON N6A 5B9 Canada; 4 Arthur Labatt Family School of Nursing, Western University, FIMS & Nursing Building, Room 3306, London, ON N6A 5B9 Canada

**Keywords:** Aboriginal, Indigenous, Marginalized populations, Underserved populations

## Abstract

**Background:**

Non-government, not-for-profit community health centres (CHCs) play a crucial role within healthcare systems in fostering equity, acting both as direct providers of services and as sentinels of health and social inequity. In a study of an intervention to promote equity-oriented health care, we enlisted four diverse primary healthcare clinics with mandates to serve highly marginalized populations. All of these CHCs operate as not-for-profit, non-government organizations (NGOs), and have a marginal relationship financially and socially to other parts of the system. The purpose of this paper is to provide an analysis of the factors that shape how CHCs are able to carry out an equity mandate and, from this, to identify what is required at the level of policy to enhance capacity to provide equity-oriented health care.

**Methods:**

We systematically examined the clinics’ policy and funding contexts, and identified influences on the clinics’ capacities to promote equity-oriented health care.

**Results:**

We identified three key mechanisms of influence, each playing out against the backdrop of a contested and marginal position of CHCs within the health care system: a) accountability and performance frameworks; b) patterns of funding and allocation of resources, and c) pathways for emergent priorities. We examine these mechanisms, considering how each influenced the pursuit of equity, and propose policy directions to optimize the primary health care sectors’ capacity to support equity-oriented health care.

**Conclusions:**

Although this analysis is based on a study within a high-income country, we argue that because the dynamics between community health centres and broader healthcare systems are similar across national boundaries, the implications have applicability to low and middle-income countries.

## Background

Healthcare systems are composed of multiple organizations (Health Departments, Regional Health Authorities, hospitals, independent for-profit services providers, corporations and non-government not-for-profit organizations), each with its own governance structure, priorities, accountabilities, mandates and budgetary constraints. Legislation, policies and contractual obligations link these organizations together to create a system where discontinuities of care can become visible and hopefully remedied. Where these links are lacking, relationships that depend on the will of individuals can act as short-lived patches across gaps in the system.

In both higher, and lower and middle income countries (referred to as HIC and LMIC respectively), two problems persist: i) inverse care (that is, those who are most Marginalized^1^ and have the greatest health problems have the least access to care), and ii) fragmentation and under-resourcing of care for marginalized populations [2]. In most healthcare systems, non-government not-for-profit organizations (NGOs) such as community health centres (CHCs) fill service gaps left by other providers. The Canadian Association of Community Health Centers (2016) defines CHCs as “multi-sector health and healthcare organizations that deliver integrated, people-centred services and programs that reflect the needs and priorities of the diverse communities they serve. A Community Health Centre is any not-for-profit corporation, co-operative, or government agency which adheres to all five of the following domains:

1. Provides interprofessional primary care.
2. Integrates services/programs in primary care, health promotion, and community wellbeing.
3. Is community-centred.
4. Actively addresses the social determinants of health.
5. Demonstrates commitment to health equity and social justice.

While we agree that CHCs could possibly be government agencies, the CHCs with which we worked emerged as a result of government services’ failure to meet the needs of vulnerable populations [3–5]. A key component of the success of CHCs in meeting needs has been attributed to their community-grounded governance structure, which ensures pathways for community feedback on the performance of the CHCs and their programs to meet community needs [6–8]. Other studies have shown that CHCs that become government agencies eventually lose some of their connection to community needs [9–11].

In LMIC, CHCs are remarkably diverse: they span the for-profit to non-profit continuum, can be religious or secular, may offer a narrow or broad scope of services, may be larger or quite small, may employ professionals or depend on volunteer, may exist to fulfil short or long term objectives, and may be single or multisector focused [12]. Funding for their existence may be from international secular or faith-based organizations, from philanthropic foundations, from membership, and/or a combinations of these. In their scoping review of non-governmental organization’s contribution to global health, Anbazhagan and Surekha highlighted the following strengths: great variety of programs to meet local needs; flexible and agile to ensure a quick response to emerging needs; generally have low operating costs; depend on staff and/or volunteers with a high level of commitment; community-embedded; often less tainted by association with the local or national government; and less likely to fall to corruption [12]. Their distancing from local and national government also means that their accountability to the national health authorities vary [13–15]: this can be a strength or a concern, depending on context.

In HIC, CHCs emerge out of two separate processes. In some cases, national and/or regional healthcare authorities promote the creation of CHCs to take on predefined tasks: services for a selected population such as Indigenous populations in Canada, Australia and New Zealand [16, 17] or selected services such as HIV counselling [18]. The push for smaller government and interest in harnessing competition among providers to stimulate innovation can act as an added incentive for governments to transfer healthcare responsibilities onto the NGO sector [19]. In other cases, however, CHCs surface primarily in urban areas where surplus capacity (underemployed community-engaged professionals) exists and where unmet needs persist [5], to complement services provided by for-profit providers (providers in private practice) and governmental not-for-profit providers. Although histories vary, in either case, these CHCs generally emerge as a result of social activism, to meet the unique, and unmet or poorly met, healthcare needs of marginalized populations.

We argue that CHCs in HIC and LMIC implicitly or explicitly operate with an equity mandate, leading them to prioritize the development of services and programs that can best improve the health and social care needs of the population(s) they serve. However, as we discuss in this paper, the capacity to provide health care that is equity-oriented can be undermined by policy and funding contexts. In resource-stretched healthcare systems, these factors are important to understand and address.

The CHCs we engaged with for this study were funded primarily through public money and occupy a potentially ambiguous place in the healthcare systems, as they operate semi-autonomously. We have observed the same situation in other CHCs across Canada, Colombia, Australia and New Zealand [5, 20–24]. Whereas government employees can be directly controlled through firing and other mechanisms in government-owned and operated services, governmentfunders hold CHCs accountable for their use of public finances through accountability frameworks focused on contractually defined outputs and outcomes [8, 25]. However these may lead to tension between the CHCs and their funders: CHCs’ accountability to their service communities often leads them to advocate for those communities to government agencies, which may themselves be the CHCs’ funders [5, 8] or the policy-makers who create or prune the space CHCs can occupy. Funders who can also be service providers, have a dual role in this dynamic: they can be both the source of unmet needs (since the services they provide are failing to meet these needs) and the solution (through their funding of CHCs, albeit at modest levels compared to government delivered services). Little is known about how to create a policy-entrenched environment that would support CHCs in creating and operationalizing effective and equity-informed health services, when the policy-makers are also those who may be falling short of meeting needs. A detailed understanding of the factors enabling and constraining the capacity of CHCs to enact an equity mandate would optimize the effectiveness of their advocacy roles and aid funders and policy makers in designing better approaches.

This paper discusses policy implications stemming from a program of research examining an innovative primary healthcare (PHC) intervention – EQUIP Primary Healthcare – designed to enhance capacity for equity-oriented health care (EOHC) at PHC clinics serving marginalized populations [1, 26–29]. One aspect of this research focuses on the policy environment required to support EOHC, defined as care that aims to mitigate the negative health effects of structural inequities and structural violence [1]. Our work has contributed to international conversations focused on the provision of trauma- and violence- informed, culturally safe and equitable PHC services to populations affected by health inequities and marginalizing social conditions [30]. In this paper, we identify the policy level requirements to expand the capacity for equity within healthcare systems, including enhancing the role of CHCs as “*sentinels of inequity*”. We highlight key mechanisms that facilitate or impede the ability of CHCs to realise their equity mandate, and illustrate how these mechanisms operate in the context of four Canadian CHCs that provide PHC for populations living in marginalizing conditions, who are generally underserved by other sectors of the healthcare system. These findings have implications for improving equity across Canada’s healthcare systems and have relevance more widely.

## Theoretical foundations

The CHCs discussed in this study are not-for-profit NGOs. A large body of literature, primarily from the field of economics, focuses on the role the NGO sector plays in the provision of public goods. Of relevance, the theoretical literature offers explanations as to why NGOs emerge to provide public goods in parallel to public institutions, and how these organizations are positioned in relation to public institutions. Salamon and Anheier [31] noted six broad theories that aim to explain this phenomenon, briefly described as follows. Weisbrod’s *government failure or heterogeneity theory* [10, 11] suggests that government provision of public goods becomes homogenous over time in response to the needs of the majority. The NGO sector emerges to meet unmet needs: it is a response to demand for both enhanced quantity and range of public goods. Thus, this theory implies that NGOs are created to provide goods for populations whose needs and preferences are not met by government and other providers (e.g. through family physicians in private practice). Heterogeneity of needs and preferences is thus key. Somewhat interrelated is Hansmann’s *trust theory* [32] that posits that information asymmetries between providers and consumers create distrust about the quality of the product being procured. NGOs, being by definition not-for-profit, may be (or at least may be assumed to be) more trustworthy. Thus, distrust in government providers or markets is key to the emergence of NGOs.

James’ *supply-side theory* [33] suggests demand alone is not sufficient, and that social entrepreneurs must exist in order for NGOs to emerge. James argues that this is particularly true in areas where religious competition exists, resulting in religious-based NGOs organizing for the delivery of public goods as a mechanism to attract adherents. The surplus delivery capacity that exists in many urban centres can also explain the proliferation of NGOs providing social goods to underserved populations. In their discussion of *welfare state theory*, Salamon and Anheier [34] suggest that the literature treats the NGO sector as a residue from imperfectly developed welfare states. Thus, it is assumed that greater involvement of the state in social welfare services leads to a smaller NGO sector. Finally, Valentinov [35] developed a *rurality theory*, arguing that NGOs emerge in rural settings to bridge inequities in services experienced in those areas.

The theories discussed above tend to position the state as in conflict with the NGO sector. In contrast, the *interdependence theory* suggests that conflict coexists with interdependence and partnership [34]. For example, the NGO sector can be mobilized more readily than government, and can secure political support to ensure that government engagement occurs in areas of public interest. Salamon and Anheier further argue that, along with government and market failure, “NGO failure” also exists in that NGOs depend, to a greater or lesser degree, on government funding for their existence. Finally, they articulate a *social origins theory* that recognizes that NGOs do not “float freely in social space” ([34], p. 18.) but are rather products of, and embedded in, social and economic structures.

These theoretical perspectives suggest that NGOs have a unique and valuable role to play in the pursuit of transforming health systems towards increased equity. Although helpful lens, they focus on the mergence of NGOs but fail to adequately position NGOs within the healthcare system in which they operate. It is our observation that government-funders have an ambiguous relationship with NGOs, including CHCs. Government-funders promote CHCs as a viable extension of healthcare systems, recognizing their unique role in the provision of needed services. At the same time, CHCs are not simply extensions of government-funded services, since they often fall outside traditional accountability relationships. This autonomy can be framed as being at odds with the push to increase accountability across healthcare systems. New systems of accountability have shifted from being based on trust, to those that emphasize oversight and control [16, 36], which can undermine the sustainability and equity contributions of the NGO sector [6, 8], for example by setting onerous reporting requirements, setting targets for specific screening tests or requiring measurement against certain indicators in ways that shape practice toward such activities rather than the most pressing patient needs.

Our program of research is informed by an equity lens, based on an understanding that all policy is value laden and all policy work, since it involves decisions that affect populations who have little involvement in policy decision-making [37], is ethical work. In developing this lens, we drew upon intersectional theory to guide our examination of how different forms of structural oppression are constructed, affecting individuals, organizations, and broader social systems in complex and interdependent ways. Because intersectional scholarship, driven by the pursuit of social justice [38], is oriented beyond descriptive analyses toward eradicating inequities, it provides an ideal foundation for work aimed at moving policy towards equity. An equity lens directs attention to the role of policy in shaping the relationship between social conditions and health inequities, and related health system responsibilities, in four key areas: 1) the contexts and conditions that shape access to healthcare and health outcomes; 2) structural determinants of health; 3) a wide range of evidence related to social, historical and cultural roots of disadvantage; and 4) distribution of power in both the production of inequities and policy processes (Pauly B, Varcoe C, McPherson G, Laliberté S, Reimer J, Ponic P, Hancock T, Kenny N: Conceptualizing an equity lens for public health: The contribution of health policy ethics, in review). In this paper, we apply an equity lens to examine the policy and funding contexts of four diverse CHCs. We draw on interdependence theory to frame the role of the CHCs as being that of *sentinels of inequity*,^2^ *leading* the development of responses to current and emerging unmet needs, *advocating* to other service providers and health departments, and *educating* the healthcare system.

There are significant gaps in knowledge concerning how to: make services as responsive as possible for marginalized populations; address the health effects of structural inequities and structural violence; make PHC service delivery reforms more socially relevant; and create policy and funding environments to support these aims [39]. Our analysis considers the latter: how might policy decisions better promote EOHC throughout healthcare systems through supporting CHCs? We sought to understand how contemporary and emerging political climates shape equity agendas and to identify opportunities to enhance equity both locally and across sites.

## Methods

This analysis was conducted as part of a larger study, entitled EQUIP Primary Healthcare, aimed at developing new knowledge regarding how PHC services can serve as a key pathway to health equity at a population level. The study received ethical approval from the Behavioral Research Ethics Boards of the University of British Columbia (H12–02994) and The University of Western Ontario (103357). All participants completed an informed consent process.

Using a mixed methods, multiple case study design within a participatory framework, we developed and implemented an equity-oriented intervention in four PHC clinics and examined changes in key outcomes for patients, staff and the organizations. Drawing on multiple sources of data, case study method is suited to studying the structures and processes of bounded systems in context. In multiple case study, the emphasis is placed on generating both a detailed understanding of each case *and* a broader understanding of commonalities that exist across cases [1]. In our study, the PHC clinics served as the cases. We interpreted the impacts of the intervention within a broad understanding of the process of implementation at each site, including how the policy context shaped their ability to implement and/or improve their equity-oriented models of care.

For the analysis presented in this paper, three types of data were used. First, we developed a socio-historical narrative regarding each clinic, detailing its history, the history of the communities each clinic served, and its contemporary profile, including its position within the wider healthcare system. To construct these profiles, we drew on the clinic’s own historical and current records, and historical accounts of the communities. These narratives were used in multiple ways, including as a tool to build consensus among different stakeholders (e.g., between staff and community organizations, staff and board members) as to the history of, and challenges facing, the clinics. Second, following a method developed by Lavoie [3], we reviewed and analyzed the minutes of Board meetings along with funding contracts for each clinic for a 5-year period (2011–2016) to construct a profile of each clinic’s contractual environment. Minutes were reviewed to identify internal pressures (e.g. lack of resources, relationship with staff, tensions) and external pressures (e.g. new policies, relationship with funders and the community, emerging unmet needs). Contracts were analyzed to assess funding stability over time, budgetary line flexibility, alignment between contractual obligations and the clinics’ day-to-day operation, as well as reporting burden. Third, we conducted in-depth interviews with leaders (administrative and clinical leads, Board members) at each of the clinics (*n* = 7) specifically focused on the policy/funding context and its impact on the clinic. These interviews were audio recorded and transcribed verbatim.

To analyze this data, the team assigned one research lead per CHC to collate the information available and conduct a preliminary analysis of Board minutes and financial records and contracts. Missing documents and gaps in knowledge were systematically identified, and shared with the team (the four leads) in regular meetings. Once major gaps were filled, focused interviews were conducted to address any remaining gaps in knowledge. A cross-case analysis was then undertaken to identify commonalties and differences.

## Results

The CHCs we studied emerged in areas with unmet PHC needs. Importantly, these needs were unmet because the people being underserved faced complex challenges in terms of both their health status, socioeconomic conditions, and their ability to access quality and responsive healthcare. The particular unmet needs to which they responded initially set the course for each clinic’s development. Each of the four CHCs in our study arose through their own unique sets of circumstances, needs and opportunities, and as shown in Table 1, have been described in more detail in a prior publication [40]. The patient populations served by these clinics ranged in size from 1300 to 3700 individuals.

**Table 1:** Descriptions of Each Clinic

Organizational Features	
Clinic W^a^	• Founded in 2011. • Located in a city which is a regional hub for many rural communities. • Serves people who face barriers to health and social care and those ‘in transition,’ with a primary focus on women and families living in marginalizing conditions, including recent immigrants, many of whom have experienced violence and trauma. • Primary health care services include identification, ongoing assessment and management of acute and chronic health problems, counseling, education and health promotion, and support in navigating complex systems.
Clinic X	• Founded in 1994. • Located in a rural region serving rural farming communities and First Nations communities. • Provides primary care at multiple sites to populations across the lifespan, from seniors to families with young children, through direct primary care and a wide range of responsive health promotion programs.
Clinic Y	• Founded in 1991. • Located in a northern regional city where high proportions of Indigenous people reside. • Serves Indigenous and non-Indigenous people experiencing major socioeconomic challenges including people living on very low incomes, in unstable or temporary housing, and those who are unable to work due to disability. 75% of the patient population self-identifies as Indigenous. • Provides a wide range of primary health care services including medical and nursing care, counselling, social work, physiotherapy, and outreach services.
Clinic Z	• Founded 1970. • Located in an inner-city metropolis and serves low income populations, including many experiencing inadequate housing or homelessness, major mental health and substance use issues, and significant barriers to accessing basic health services. • Provides a wide range of primary health care services, including a pharmacy, dental clinic, and physical and mental health services.

^a^To protect anonymity, the clinics are designated “W, X, Y, Z”, and the interviewees are designated as being from Clinic A, B, C, D without correspondence between the two designations (thus obscuring which clinic each interviewee was from).

Our findings show that CHCs which have a close relationship with their funder, and whose mandate in the overall healthcare system is clearer (for example, Clinic Y), benefit from a more flexible funding environment and adapted accountability frameworks. Where distance and ambiguity exist, CHCs’ contributions to equity is undermined.

[W]hen there’s a request to do something that all the other primary care organizations do, the request isn’t always filtered through an understanding of ‘would this need to be different for the [CHC]?’ Until we come back and go ‘wait a minute guys this doesn’t make any sense’… so just, you know, as an example, we all report on the same indicators every year. (Clinic B-C03)

In my opinion [the funders] still do not have an understanding of the value of the CHCs on the system. They know we do some things, they know we work with [the] vulnerable, but it’s not, it’s not on the same respect or level of the other [fee-for-service] bigger model which is maybe to be expected because you’ve got ten thousand [fee-for-service] doctors and you’ve got two hundred [CHC-based] doctors. (Clinic D-C01)

We identified three key mechanisms shaping the provision of equity-oriented care that can potentially undermine CHCs’ equity mandate: the use of accountability metrics/indicators that are not matched to an equity mandate, patterns of funding and allocation of resources that are poorly tailored to needs, and the lack of support for continuous change management. These mechanisms persist because of a lack of clarity regarding the role that CHCs ought, and can, play, in an equity-oriented healthcare system.

### Accountability and performance frameworks are not matched to an equity mandate

In an equity-oriented healthcare system, where different populations are served by a variety of service providers (e.g. fee-for-service family physicians, salaried nurse practitioners, CHCs), indicators of performance monitoring must reflect the populations served, and be benchmarked appropriately to reflect patient complexities. The CHCs we studied, however, reported that the performance indicators they report on fail to appropriately or completely reflect their population’s needs and services provided.

[S]o we’ve been operating this, this service for X amount of years and then all of a sudden the contract this year stipulates these different [performance indicators], you know, in terms of measurements… it’s just all of a sudden they’re going to start measuring these indicators so I mean we’ll see how that goes. (Clinic C-C01)

Even in cases where there were efforts to index funding to complexity, the alignment was not ideal.

Yeah so [some CHCs] have a complexity score that’s used to adjust their panel size [number of patients the clinic is expected to serve], you know, they would say it isn’t perfect, in fact it has a lot of weaknesses in it but it’s better than not having anything. So there also has been some discussion about using that type of a scoring mechanism with [our clinic] to adjust our… panel size or an expectation based on complexity which for [our clinic] would be a helpful thing because we’re a bit of an odd duck. (Clinic B-C03)

Importantly, when used, these efforts were typically only used to estimate numbers of patients to be served, not what targets are set or indicators used. For example, a clinic serving primarily women with children who have experienced high levels of violence, poverty and homelessness, and a clinic primarily serving a high-income area both must achieve 70% Pap smear targets.

CHCs’ ability to meet their benchmarks is contingent on funding, staffing and infrastructure adequately aligned to the needs of the population served. Short-term funding contracts, and an inability to offer competitive salary and benefits, can result in staff attrition and undermine the CHCs’ ability to meet their contractual obligations.

So they don’t ever take that into consideration that, you know, the physicians - we need to be fully staffed and if you don’t have the right recruitment or salaries, that hard. And if you don’t have that then you can’t reach panel size. If you don’t have infrastructure … like you’re supposed to have three examining rooms for each provider, we have one. You’re supposed to have so many nurses for a thing, we only have one right, you know, so you don’t have the supports or the infrastructure [and] that will reduce your ability to meet panel size, right? (Clinic D-C01)

Unique features of equity-oriented CHCs, such as culturally-safe care, and trauma- and violence-informed care, are not captured by conventional indicators.

[The] reporting, you know, this has been an area of tension particularly in the health center is the things that we think are important in terms of health metrics are very different than the things that our contract agencies think are [important] in terms of metrics. I mean obviously we think access, you know, they’re very focused on how again the number, how many people are receiving these types of things… I mean some of the health outcomes which are very easy to identify in terms of Hep C or HIV, viral suppression in clients and things like that but some of the other things that we think are important aren’t really that important to them. So there is that tension there in terms of we’re trying to deliver a certain set of values and services and the funders … sometimes [their priorities are] different. (Clinic C-C01)

Throughout the study, the clinics sought to identify, integrate and monitor indicators that were reflective of their equity mandate, including those for acceptability, accessibility and safety for people marginalized by poverty and multiple forms of discrimination, how well they were able to facilitate access to health services and social determinants, and overall fit of care to needs. These are however not the indicators embedded in the accountability frameworks used by funders. Thus, the essential work performed by the clinics remain largely invisible, and undervalued.

### The patterns of funding and allocation of resources are not optimally tailored to needs

CHCs perform best when able to tailor services to those they serve. In an equity-oriented healthcare system, they require resources to support their ability to respond to ever-changing needs.

The CHCs we studied access funding through streamlined and sustainable sources (Clinic Y), or fragmented and siloed but generally renewable funding (Clinic Z, Clinic X), or a constellation of yearly contracts with specific performance expectations (Clinic W). With the exception of Clinic Y, all reported considerable misalignment between their funding, the size and needs of the population they serve, and serious gaps in funding in key areas, limiting their ability to respond to existing and emerging needs.

[T]he original funding formula for [our CHC] has not changed in twenty years, it’s been a little bit modified with the new [Ministry mandate], a few different positions. But the original core, particularly the primary care nurse practitioner and physician formula that you get from the ministry, you know, I have the same number of doctors here as [when] I started twenty-one years ago. (Clinic D-C01)

[I]t looks to me like that’s the budget we’ve always had, that’s the same budget we’ve had by the way from the time we were established, there’s been no increases… [T]he salaries we have to pay to the staff are set by the Ministry, we have a little bit of a range and we’ve tried to set criteria to be fair to people who’ve got more experience or less. We couldn’t deal with that so what we’ve chosen to do in a policy way is to give some people more vacation time or something to at least reward them. It’s not a nice system but the system itself we had to work within what the Board had the availability to do. (Clinic B-C02)

Flexibility has been eroded where it once existed. In some clinics, flexibility is based on long term, trust-based relationships between funders and clinic administrators. These relationships can however be eroded as a result of attrition (either with the clinic, the funder or both).

And I think we’ve always had a really good working relationship with the person assigned to manage our contract. And actually a week ago, the first time, the earliest ever we had a meeting with our funding person and so it was, they really wanted to get out in front of the budget process of [our main funder] to make sure that if anything happened within these strained times that there might be something they can float our way in terms of assisting our growth. (Clinic A-C01)

[W]e had to get rezoning for two of the clinics from the city. So we ended up, we had a conversation with our Ministry person at the time who said *don’t worry* about it just go ahead and do it. And, of course, that person changed… you see where I’m going with this? So it’s been an ongoing discussion with the Ministry about how do we settle up and finally last year … after three plus years [we] finally got sorted out how much money was actually due back to the Ministry… And so last year finally that big cheque was the money that had been sitting in our bank account that we knew had to be returned, some of it, we didn’t know the amount, finally got sorted out. (Clinic B-C03)

Opportunities to innovate to ensure continued responsiveness to the community’s needs are at times thwarted by siloed thinking on the part of funders, and the lack of value attached to CHCs. For example, one CHC was approached by a family physician who wanted to retire and who wanted the CHC to take over his caseload, a significant number of whom had complex health issues and challenges accessing health care. Initial meetings with the CHC’s primary government funder, who also, through the provincial health insurance plan, funds family physicians being paid on a fee-for-service basis, seemed promising. However, after over a year of discussions, including the CHC developing a business plan, the funder decided to transfer the funding to another fee-for-service based provider group:

So why is the [CHC] not an option, why are we just thrown away again and the [family health team] will get the money because that’s what they always do? (Clinic B-C01)

But we, we become I don’t know jaded or whatever you want to call it over the years when, you know, the community money that’s to come to the [primary funder] goes to the [hospitals] each year, right? Like they get about five million and its always a priority, [the hospital] is always in deficit but … are there’s probably ten, twenty of us [CHCs] that will be in deficit this year, the older centers. (Clinic B-C01)

All the CHCs we studied received the vast majority of their funding from government agencies (over 90%). While unallocated funding under primary contracts must generally be returned to the funder at year-end, CHCs are responsible for any deficits. Opportunities exist to seek alternative funding sources such as competing for project dollars or fundraising, but all recognized this as problematic.

[W]e had made some decisions several years ago that we weren’t going to be forced to be in competition with our, with our colleagues and with our sister organizations. (Clinic A-C01)

CHCs might collaborate with other NGOs to decide which agency is best positioned to apply for funding and eliminate opportunities for funders to use competition. While competition is not necessarily problematic, it does pit NGOs against NGOs and can undermine relationships among those who also must collaborate to ensure that community members can access a broad range of services that fit their needs. A competition-based process also leads NGOs, and in this case CHCs, to spend considerable time on writing proposals that may not be funded. Finally, many calls for proposals relevant to NGOs and CHCs are for relatively small pots of funding.

[W]e’re very deliberately not looking for a lot of proposals or one time funding due to our [in]ability to manage that. So every little additional funding comes with reporting, we’ve kind of reached our maximum on our ability to do all those reports. Nor do we like hiring people for a year and then terminating their employment… (Clinic B-C01).

I mean a lot of the other [CHCs] fundraise, a lot of the other [CHCs] have foundations, we’ve looked down that avenue a bit but we find, you know, in a little town you’re competing with the other people that need the money so that really defeats some of the community good will right? [W]e looked into the foundation this past year but we didn’t decide to go in that direction … [Also] we don’t have proposal writers or people looking at opportunities for money, if you will. So I mean… something [we] might have to focus on is trying to find some sustainable [funding], but [this] is not that easy to find, right? So I try to go more for base funding so I’ve written four or five business cases to [our funder] … just lately for an integrated chronic disease team to help with a little bit more money there. I’ve written, you know, for salaries, I’ve written for the roof, I wrote for a memory team, I can see the trend of dementia increasing and no resources for that. (Clinic B-C01)

A key need expressed by the CHCs was to access capital funding to secure and maintain facilities. Contracts that frame the funded services as essential may support the retrofitting of facilities, and provide upkeep funding. This was the case when the funders clearly saw the CHC as an extension of the local healthcare system. In contexts where this was not the case (three out of four sites in our study), fundraising was key to survival. Where fundraising is not possible (given the community served), sustainability can be compromised.

We have two people dedicated to fundraising and communications because they’re so inter-related. Right now a big focus of our fundraising efforts, though, is on our capital campaign for building housing and that’s where my time gets spent. (Clinic C-C01)

Yeah no capital reserves. I had to create four exam rooms on my own budget; like I’ve got no money for that, right? So I created more exam rooms to try to be more efficient; you just try and it’s not the best way to operate without any kind of reserve for, for maintenance... (Clinic B-C01)

### Funding mechanisms are not structured to be responsive to emergent priorities

Although each clinic was founded in response to a given set of unmet needs, the breadth and complexity of those needs has continued to expand and shift. For example, one clinic began to explicitly serve Indigenous people, but insisted on an inclusive mandate, serving all people living in poverty in the community. CHC mandates inherently mean that people who are not well served by the broader system are increasingly funneled to them.

When we actually tracked the health and social issues that these people were presenting with, on average there were between three and sixteen… [S]o one problem at a time is not going to do it for this population and you cannot separate social issues from health issues because they intertwine... And what we did actually that really caught the politicians, is we reduced ER visits by 50 % in the initial project… I mean it was, it was so easy because *they had nowhere else to go*. And they had *no physicians* who would take them on, you know, because they’re *too complicated*… And in addition to that they, they went to walk-in clinics and, of course, they have complex issues so there’s no continuity of care. And they go to ERs and they’d get *almost thrown out*. (Clinic B- C02, emphasis added)

The CHCs understand that operationalizing their mandate and equity orientation requires them to identify existing and emergent health and social conditions that create inequities in order to advocate and provide care tailored to the needs of local populations. Consequently, the populations they serve also expand and shift. In addition, they become *the* clinic serving more complex and challenging populations. This is not necessarily problematic, if matched with appropriate resourcing. One leader explained how funding flexibility allowed them to adapt to needs.

[W]e welcome persons with disabilities and we, our funding model does allow for that collaboration of team members to, to work on case conferencing and work on the best for the client. So I think we’re lucky that way… (Clinic D-C01)

Paradoxically, trust in CHCs’ ability to meet the complex needs of marginalized populations allows other service providers (fee-for-service physicians, publicly funded PHC access clinics) to redirect complex patients to CHCs, and divest themselves of the responsibility to provide care to these people. This redirection may be financially advantageous to service providers, and problematic for the CHC, unless associated with adequate resourcing. Redirection is at times also actively and explicitly pursued by government-funders, requiring CHCs to redesign programs and processes to adapt to new locales or populations (Clinic Z-001). Rarely is the redirecting bi-directional, in part because there is no incentive for family physicians and other providers to take on complex patients, and in part because the role of the CHCs in sharing how the models of care they develop can be adopted by other providers is not recognized, funded nor systematized. For example, one of our partners discussed how the introduction of a new service in an urban area near the original, rurally-based, CHC was initially intended to be a sharing of approaches that would be implemented by other providers, and how this focus shifted over time, leaving then inappropriately funded to operate multiple communities:

So we’re very small staff as you know... But it was a whole new community, it was an urban community, you know, we were from the country… so it meant a whole new relationship building with a whole bunch of new partners [and], you know, we hadn’t been part of that group before. And it came with *no additional administrative support* so that was another… so that was okay because we didn’t think we were going to be there to run it, we were just getting it going. But then when it ended up that it was put under our umbrella that was significant so we had to relook at how we operated everything, right? And the next year then [another community] came so it was back-to-back years of capital and new communities so that was significant. (Clinic D-C01, emphasis added)

In some cases, CHCs are able to refer patients to other CHCs set up to support specific populations. A key condition for this to occur is the co-existence of multiple CHCs, each serving distinct populations.

[N]ow there is a youth clinic here in the city that we encourage youth to [attend]… we certainly see, you know, a few youth here but we certainly encourage the youth to go to the youth clinic. (Clinic C-C01)

However, this is unlikely to occur in rural and even northern urban centres where surplus provider capacity is scarce.

These kinds of redirecting – to CHCs and between CHCs – or funnelling, is dynamic within constantly shifting systems and contexts, and changes as new issues become championed within the broader healthcare system (for example frail elders, dementia, diabetes, refugees) while others remain hidden or less attractive as a basis for social policy initiatives (for example, HIV transmission among people who use IV drugs, the opioid crisis).

CHCs are somewhat more agile than their counterparts that are directly embedded and financed within the healthcare system, and thus CHCs are better able to recognize emerging needs and respond. This was the case in the late 1980s in the context of the HIV crisis, and is now apparent as Canada opens its doors to a significant number of refugees. Through their community relationships, the CHCs we studied significantly expanded the healthcare system’s capacity to effectively respond to emerging needs, and were able to alert funders and governments to emergent issues. As such they play an essential, sentinel, role in any equity-oriented healthcare system.

This function can, however, be undermined by funding models that dis-incentivize taking on complex clients, or are coopted by funders who may be tempted to depend on CHCs for stopgap measures, while overlooking that CHCs can serve a key role in providing models for and educating other providers to increase the overall equity capacity of the healthcare system. Thus pathways between funding and emerging priorities tend to be reactive to crises, rather than proactive.

## Discussion

### The ambiguous and underdeveloped role of CHCs within healthcare systems

Our findings show that CHCs have an essential role to play in increasing equity in the broader healthcare system, pointing to four key roles: first, CHCs are *sentinels of inequity*. Their commitment to meeting the needs of the community makes them more likely to become aware of inequities, or emerging vulnerable populations, and of healthcare crises. Second, CHCs are better equipped to *develop care responses* that fit with the evolving needs and contexts of local populations. This is partly due to their agility (i.e., ability to respond quickly) but also to their connection with the community, which can result in community-driven or at least community-informed innovations. Once new needs have been identified, CHCs are (or at least should be) able to *advocate* to the larger healthcare system to ensure that emerging needs are recognized and met. Finally, CHCs are well equipped to *educate* the healthcare system in the development of a system-wide response to new needs. Our data also show that this last role is rarely enacted, because of lack of recognition, resources and time. Other roles remain under-operationalized.

The CHCs we studied saw their populations shift and expand, at times through choices made by the clinics (such as a recognition of the need to include people with disabilities) but often out of necessity (such as in response to an influx of newcomers, and in the absence of other health care sector responses). While they were able to develop some responses to meet emerging needs, they did so with limited resources, which strained their ability to deliver current services, and protect staff and leaders from burnout. Our analysis shows that the CHCs were not in a position to educate the broader system regarding the system-level responses to the needs of vulnerable and underserved populations. If this happened, the CHCs could play an essential transformative role in the healthcare system. This role was unrealized largely because CHCs are positioned in the healthcare system as “lesser than” formal care structures. As a result, opportunities to exchange ideas simply did not exist.

The CHCs we studied fit well within a number of theories discussed earlier in this paper, namely,

- heterogeneity theory: all 4 CHCs emerged to provide services that complemented those that existed but served specific populations poorly;
- trust theory: all 4 CHCs developed relationships with the communities they served and legitimately claimed a closer, trust-based relationship with their clients;
- supply-side theory: the urban-based CHCs were able to draw on a skilled workforce to meet their human resource needs; and
- welfare state and rural theories: all were filling important service gaps left by government-sponsored services.

While a good explanatory fit, these theoretical framings provide little direction on how to operationalize and promote CHCs’ equity role in healthcare systems. Salamon’s interdependency theory [41] offers some promise. Three decades ago, Salamon framed the relationship between the US welfare state and the NGO sector as a partnership, where governments are best positioned to provide a steady stream of resources, set priorities, articulate high-level values for which systems must strive, and institute quality control standards. In contrast, NGOs are better able to personalize services, operate on a smaller scale, and adjust care to needs rather to the structure of government agencies ([41], p. 42). These roles are inherently incomplete in themselves, hence complementary.

More recently, Svidroňová and colleagues’ study of 60 NGOs in Slovakia concluded that interdependency theory best explains the role the NGO sector plays in the delivery of social goods [42], including the enhancement of equity. An empirical study by Lecy and van Slyke [43] which tested Weisbrod’s *government failure or heterogeneity theory* against Salamon’s *interdependency theory* found that the NGO sector is stronger when NGOs have a robust interdependent relationship with government-funders. State-NGO interdependence requires a facilitating relationship. An important body of literature has emerged reporting on the nature of this relationship (see for examples, [44–46]), but this literature remains largely descriptive and has not yet addressed the following question: *given interdependence, how should the relationship be structured to ensure that the NGO sector can achieve its equity mandate?* Brinkerhoff [47] has argued that State-NGO relationships require a high degree of mutuality, manifested by coordination, joint decision-making and mutual accountability, and the maintenance of organizational identity. This in our view requires a policy enabling environment.

It is our position that, in order for healthcare systems to fully realize CHCs’ equity potential, five policy conditions must be met:

1. System-wide recognition of the unique equity-enhancing role that CHCs play, including understanding how they complement other state-sponsored services;
2. Availability and use of performance indicators that map onto the needs and issues most salient to local populations to ensure accountability for the use of public funds;
3. Resourcing to support innovation and responsiveness to community needs, to capitalize on CHCs’ agility;
4. Feedback pathways to ensure a system-wide understanding of emerging needs; and
5. System uptake of appropriate models of care for specific populations.

We recognize some obvious limitations to our study. We studied 4 CHCs operating it the Canadian context. Although we are aware of the generalizability of our findings to other settings (elsewhere in Canada, Australia, New Zealand, Colombia, and to a lesser extent, Niger), we cannot claim generalizability across the full diversity of CHCs. We recognize that a key feature discussed in this paper is the role of the government-funder which shapes accountabilities of the CHCs. Arguably, CHCs funded through philanthropic means, by their membership, faith-based organizations or other means will face different power relations with their funders, and different forms of accountabilities. Still, we believe that CHCs operating in LMIC, which are less likely to be government-funded and therefore closely regulated, are likely be undermine by a lack of system-wide recognition, are vulnerable if unable to track their performance in the delivery of social goods, require adequate funding to deliver on their mandate, can play an invaluable role in providing up to date information to government on emerging issues, and are fertile ground for the development of innovations that might benefit other providers. We however recognize that these propositions are at this point speculative, and require testing in LMIC.

## Conclusion

The NGO sector, including the CHCs that we studied, has been shown effective at addressing inequities through innovation [48]. CHCs are an integral part of health systems design, and require a policy enabling environment to achieve their equity potential. To date, in the Canadian context, the federal and provincial governments have promoted the CHC model as key to addressing the healthcare and social care needs of vulnerable populations, and of those poorly served by other service providers, but have not provided adequate mechanisms to support CHCs in achieving this mandate.

The situation is not unique to Canada. We and others have document have similar issues in other contexts (Australia, New Zealand, Colombia). We believe that although there may be some nuances, this is also the case in many other HICs and LMIC. We believe that CHCs have an invaluable goal in the pursuit of equity, and in the pursuit of some if not all Millennium Development Goals [49].^3^ Their role must however be supported by policy. A supportive policy environment has yet to emerge. The recommendations we offer are a starting point governments and funders can use to develop a more supportive policy environment for CHCs, in the pursuit of health equity.

More research is however needed to develop better typologies of CHCs across HICs and LMICs. Further, additional work is required to refine our recommendations to ensure a good fit with this complex context.

## Abbreviations

CHC: Community health center
EOCH: Equity-oriented health care
HIC: Higher income countries
LMIC: Lower and middle income countries
NGO: Not-for-profit organization
PHC: Primary health care

## Footnotes

1 The terms ‘marginalization’ or ‘marginalized’ refer to the marginalizing conditions (social, political and economic) that create and sustain structural, social and health inequities, versus a characteristic that can be attributable to any particular population or group [1].

2 In Canada (and likely elsewhere), it was the CHCs and other NGOs who first reported the HIV epidemics in the 1980s, and who were positioned or created to respond to those affected. The same has been said of the opioid crisis.

3 which include (1) the eradication of extreme poverty and hunger; (2) the achievement universal primary education; (3) the promotion of gender equality and empower women; (4) the reduction of child mortality; (5) the improvement of maternal health; (6) combatting HIV/AIDS, malaria and other diseases; (7) ensuring environmental sustainability; and (8) developing a global partnership for development.
